# A tool for high-throughput quantification of sleep-wake transitions in data from noninvasive piezoelectric cage systems

**DOI:** 10.1016/j.crneur.2026.100156

**Published:** 2026-01-30

**Authors:** Grant S. Mannino, Andrea Lugo, Sean M. Murphy, Mark R. Opp, Rachel K. Rowe

**Affiliations:** a Department of Integrative Physiology, University of Colorado Boulder, Boulder, CO, USA; b Cumberland Biological and Ecological Research, Longmont, CO, USA

**Keywords:** Sleep-wake, Sleep fragmentation, Sleep transitions, Sleep consolidation, State transitions

## Abstract

Quantifying sleep quality in rodent models is critical for understanding its impact on neurological health and disease. Piezoelectric cage systems enable rapid, noninvasive measurement of multiple sleep metrics for large sample sizes of rodents. Although sleep duration is commonly reported, sleep fragmentation, which is a key feature of sleep architecture implicated in neurodegenerative disease, circadian rhythm disruption, and injury models, is not directly measured. We developed a standardized Microsoft Excel™-based tool for quantifying sleep-wake transitions, a scalable proxy for sleep fragmentation, in data from rodents recorded using a piezoelectric cage system. The tool extracts transitions from 2-s binned activity data, which are output by the system's software. Our pipeline, which incorporates this tool, enables high-throughput analysis of sleep fragmentation across large datasets with minimal user intervention. We demonstrate the applicability of this tool and the associated pipeline by analyzing 24-h sleep-wake behavior in wild-type male and female mice. The approach facilitated identification of biologically meaningful sex differences in sleep fragmentation patterns. Female mice exhibited more frequent transitions between sleep and wake states, particularly during the light period, consistent with increased sleep fragmentation. This is the first method developed for quantifying sleep fragmentation from activity data recorded by noninvasive piezoelectric cage systems, and represents a standardized, reproducible, and publicly accessible approach with wide application in rodent models. It enhances the utility of piezoelectric cage systems and supports noninvasive phenotyping of sleep architecture in neuroscience research, particularly where high-throughput or minimally invasive methods are required.

## Highlights

• Sleep–wake transitions quantify fragmentation with high temporal resolution.
• GLMM validation confirms exceptional consistency across multiple cohorts.
• Open-source macro enables rapid, reproducible transition quantification.
• Sex differences in sleep fragmentation were detected using this transition-based method.
• Framework adaptable to other noninvasive sleep-monitoring systems.

## Graphical abstract

**Figure:**
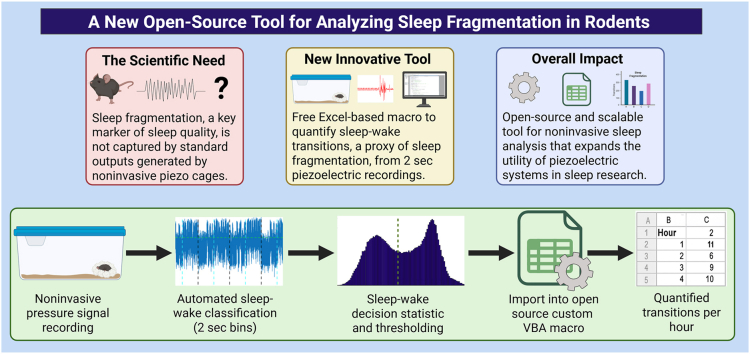

## 1 Introduction

Characterizing sleep-wake behavior is essential for understanding its impact on health and disease (Irwin, 2015). In humans, gold-standard polysomnography (PSG) assesses sleep by recording physiological parameters, including electroencephalogram (EEG), electromyogram (EMG), electrooculogram, pulse oximetry, airflow, and respiratory effort (Sadeh, 2015; Kloefkorn et al., 2020). In contrast, preclinical rodent studies typically rely on EEG/EMG electrodes (hereafter referred to as invasive) or noninvasive approaches, such as video analysis or modified cage systems (Rayan et al., 2024; Yaghouby et al., 2016; Mannino et al., 2024; Fischer et al., 2021).

Although EEG/EMG recordings are regarded as the most accurate method of determining rodent sleep, application can be limited by the required surgical implantation of recording electrodes. The surgery and implantation can cause inflammation, prolonged recovery, and occupy limited skull space, which can confound metrics of interest in some studies (Yaghouby et al., 2016; Mang and Franken, 2012; Sare et al., 2018; Balzekas et al., 2016; Spagnoli et al., 2024). Additionally, the necessary expertise and specialized equipment for EEG/EMG-based studies can be restrictive in experimental designs, and logistical and financial constraints of the method typically limit studies to small sample sizes, often resulting in low-powered studies with small effect sizes.

Noninvasive methods can serve as valuable alternatives for determining sleep-wake behavior in animal models when invasive methods are infeasible or where noninvasive approaches are preferred. Available noninvasive methods typically rely on measuring physiological parameters that correlate with sleep-wake brain states to assess rodent sleep behavior. For instance, recordings of gross body movements and respiration using force sensors, electric field sensors, or video-based scoring criteria are common (Kloefkorn et al., 2020; Sare et al., 2018; Mang et al., 2014; Geuther et al., 2022). More recent work has demonstrated the potential for differentiating all three vigilance states—rapid eye movement (REM) sleep, non-REM (NREM) sleep, and wakefulness (WAKE)—from sleep-wake data recorded using noninvasive methods (Mannino et al., 2025). Three-state sleep staging facilitates comparability between noninvasive and invasive studies by providing insights into sleep architecture, including the structure and pattern of sleep-wake cycles (Kloefkorn et al., 2020; Yaghouby et al., 2016; Mannino et al., 2024; Bastianini et al., 2017).

Despite advances in noninvasive methods, these approaches remain somewhat limited because they primarily only yield duration-based sleep metrics (e.g., total minutes of sleep (Rayan et al., 2024)). Although valuable, such metrics provide a partial understanding of sleep quality and do not reflect a comprehensive view of sleep architecture. Nevertheless, sleep has gained prominence as an experimental outcome measure in many areas of neuroscience and physiology, and advancements in noninvasive monitoring systems have increased utility in sleep research (Wang et al., 2023; Worley, 2018). There is a growing need for noninvasive, scalable methods that can capture additional dimensions of sleep architecture, particularly sleep continuity and fragmentation, in preclinical neuroscience models.

A key advantage of EEG-based methods is the ability to assess sleep continuity, as well as the duration of and transitions between specific sleep stages. These aspects of sleep architecture are critical for understanding the mechanisms underlying health and disease. For example, disruptions in sleep continuity, such as sleep fragmentation, are often associated with insomnia, one of the most common sleep disorders (Irwin and Opp, 2017). Sleep fragmentation is also increasingly implicated in neurodegenerative diseases, traumatic brain injury, and circadian misalignment, highlighting its relevance in neuroscience research. The degree of fragmentation can be quantified by simply counting sleep-wake transitions, providing valuable insight into sleep quality, a major dimension of sleep and a potential prognostic biomarker for disease. Therefore, analyzing sleep-wake transitions in preclinical studies is essential for advancing our understanding of sleep disorders and their broader health implications.

EEG-based studies often analyze sleep in ‘bouts’ or ‘epochs’, but the criteria for these terms vary among research groups due to differing methodological approaches. Because these definitions influence how sleep architecture is determined or scored, interpreting and comparing results across rodent studies can be challenging or entirely precluded. In contrast, sleep-wake transitions can be quantified in EEG-based analyses without relying on subjective sleep architecture criteria (Sutton and Opp, 2014). However, no methods currently exist to quantify sleep-wake transitions as proxies for sleep fragmentation from activity data measured with noninvasive methods, such as piezoelectric cage systems.

Herein, we describe a novel tool and associated data processing approach that can be used to objectively determine sleep behavior and quantify sleep-wake transitions from rodent activity data recorded noninvasively by a piezoelectric cage system. The method builds upon validated piezoelectric signal classification and introduces a standardized, automated workflow to quantify transitions at high temporal resolution. As a validated alternative to invasive EEG/EMG recording, the piezoelectric cage system achieves >90% accuracy in identifying sleep and wake states compared to human-scored EEG data, thereby enabling rapid, high-resolution measurement of sleep-related metrics for large sample sizes of rodents (Mang et al., 2014; Donohue et al., 2008). Our method leverages the ability of the piezoelectric system to detect sleep and wake states at 2-s intervals and integrates a custom, open-source macro for efficient quantification of transitions across large datasets. This refinement offers a practical solution for characterizing sleep fragmentation in preclinical studies, particularly when EEG/EMG recordings are infeasible or impractical.

We provide detailed guidance on data quality control, threshold-based classification, and transition detection, allowing researchers to systematically assess sleep fragmentation without relying on invasive surgical procedures or subjective post hoc annotation. To demonstrate the utility of the approach, we applied it to analyze 24-h sleep-wake behavior in wild-type male and female mice, revealing sex-specific differences in fragmentation patterns across the light-dark cycle. These findings replicate known sex differences in sleep architecture and validate the approach under normal physiological conditions. The approach is broadly applicable across neuroscience domains, including studies of aging, neurodegeneration, injury, and circadian biology.

## 2 Methods

### 2.1 Piezoelectric framework

**Fig. 1:**
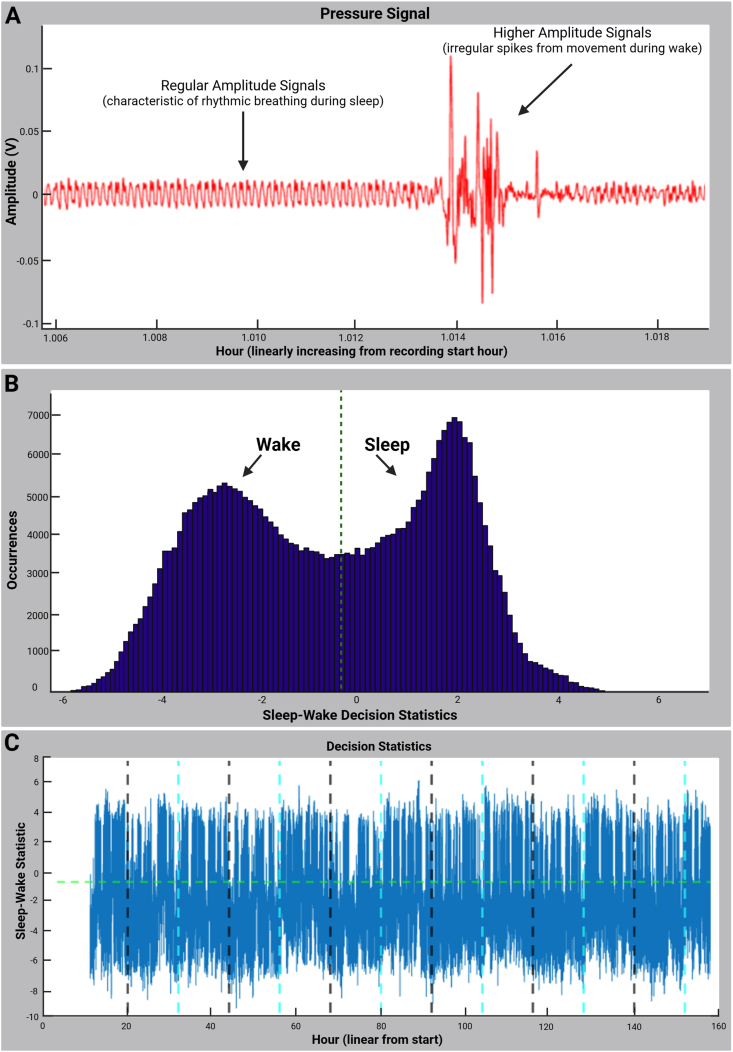
**Piezoelectric methods for distinguishing sleep and wake states utilizing decision statistics. (A)** Piezoelectric pressure signals distinguishing sleep and wake states in mice**.** The noninvasive piezoelectric cage system records pressure signals from the cage floor, measured in amplitude (V) over time. Regular amplitude signals (∼3 Hz) indicate rhythmic breathing during sleep, whereas wakefulness is characterized by higher amplitude, irregular spikes associated with volitional movement. These signals are processed using PiezoSleep software to determine sleep-wake states. **(B)** Histogram of sleep-wake decision statistics from a 24-h recording period. The sleep-wake decision statistic, derived from piezoelectric pressure signals every 2 s is plotted. The bimodal distribution reflects distinct sleep and wake states. The green vertical dashed line indicates the sleep-wake decision threshold, which is automatically calculated to maximize separation between sleep (right of the threshold; higher value than the threshold) and wakefulness (left of the threshold; lower value than the threshold). A well-separated bimodal distribution suggests effective classification of sleep and wake states. **(C)** Time series of sleep-wake decision statistics over the recording period. The sleep-wake decision statistic, calculated every 2 s, is plotted in blue over time. The x-axis represents hours from the start of the recording and increases linearly. The black vertical dashed lines indicate dark onset times, while the cyan vertical dashed lines mark light onset times. The green horizontal dashed line represents the sleep-wake decision threshold, which is used to classify each 2-s interval as either sleep (above the threshold) or wakefulness (below the threshold). This visualization allows for the assessment of sleep-wake patterns across the full recording period.

The noninvasive piezoelectric cage system (Signal Solutions, Lexington, KY, USA) determines sleep-wake behavior by coupling a polyvinylidene difluoride sensor on the floor of each cage to an input differential amplifier, which records pressure signals (Donohue et al., 2008). In rodents, regular breathing movements during quiescence generate low-amplitude (∼3 Hz) signals characteristic of sleep, whereas wakefulness is marked by higher-amplitude, irregular spikes associated with volitional movement (Fig. 1A) (Donohue et al., 2008). Identifying sleep-wake transitions requires using the sleep-wake decision statistic and decision threshold features in SleepStats Data Explorer Version 4 (Ouellette and Donohue, 2023). These features form the core analytical framework of the piezoelectric system. This section details the function of the sleep-wake decision statistic and describes how to assess accuracy of the decision statistic and decision threshold for a rodent before quantifying sleep-wake transitions.

The sleep-wake decision statistic is a measure of the likelihood of sleep or wakefulness based on features derived from the animal's pressure signals with a 2-s resolution. This measure incorporates signal amplitude, periodicity, and regularity (Yaghouby et al., 2016). A more positive value indicates a higher likelihood that the animal was asleep during that 2-s interval. In the SleepStats program, sleep-wake decision statistics are plotted as a histogram, which should be bimodal if the statistic effectively distinguishes between sleep and wakefulness. Fig. 1B shows an example of this histogram over a 24-h recording period (43,200 2-s intervals). The green vertical dashed line represents the sleep-wake decision threshold for a specific animal. This threshold is automatically calculated for each cage by maximizing the separation between sleep and wake clusters. Values to the right of the threshold correspond to sleep, whereas values to the left indicate wakefulness. If the program accurately differentiates sleep and wake states, the decision threshold should fall between the two modes.

A time series of decision statistics can also be plotted for each channel (cage) over the entire recording period, as shown in Fig. 1C. The black vertical dashed lines indicate dark onset times, whereas the cyan vertical dashed lines denote light onset times. The time axis, measured in hours, begins at the start of the recording and increases linearly. The green horizontal dashed line and the Sleep/Wake Decision Threshold on the left panel represent the sleep-wake decision threshold for the plotted channel. Each 2-s interval is classified as sleep if its decision statistic exceeds this threshold; otherwise, the interval is classified as wakefulness.

**Fig. 2:**
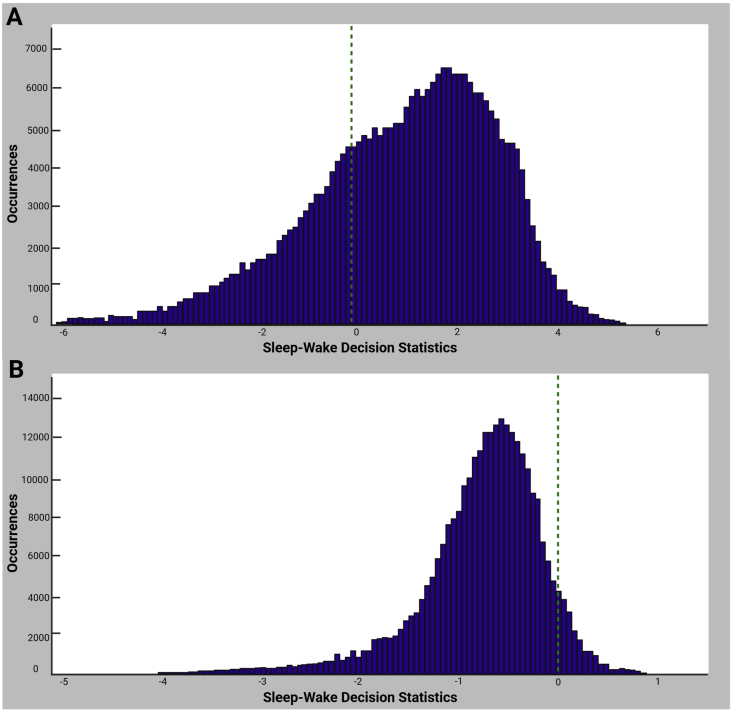
**Examples of unimodal sleep-wake decision statistic histograms indicating poor classification. (A)** A histogram displaying a unimodal distribution caused by high background noise. The lack of distinct separation between sleep and wake states suggests unreliable classification. **(B)** A histogram from an empty cage, where the decision statistics form a skewed unimodal distribution, incorrectly classifying noise as sleep. In both cases, the green vertical dashed line represents the sleep-wake decision threshold. Because the threshold does not effectively separate sleep and wake states, data from such histograms should not be used for transition calculations.

Wake behavior is often more variable than sleep, resulting in a less distinct peak on the left side of the sleep-wake decision statistic histogram. However, if the decision threshold is between the two clusters, as shown in Fig. 1B, the algorithm has effectively distinguished between sleep and wake states. If the histogram appears unimodal or the decision threshold fails to clearly separate the two behavioral states, the data should not be used to calculate transitions. This issue may arise due to high background noise (Fig. 2A) or empty cages (Fig. 2B). Although it is possible to manually adjust the threshold by inspecting the decision statistic histogram and time series, this introduces subjectivity and is not recommended.

We importantly note that the data shown in Fig. 2A and B are not from animals included in the present study but rather are provided as illustrative examples of poor signal classification. Fig. 2A shows a unimodal decision-statistic distribution caused by transient mechanical vibration that occurred when the cage was positioned near an air-handling unit. Such vibration artifacts can obscure the bimodal structure typically observed when sleep and wake states are clearly separable. Fig. 2B demonstrates a similar issue in an empty cage. These examples were included to help readers recognize and troubleshoot poor signal quality in their own datasets. In the application example in Section 3 below, all recordings were conducted on rubber vibration-isolation mats within a noise-, temperature-, and humidity-controlled environmental chamber, and all sleep-wake decision statistic histograms were verified to be bimodal prior to analysis.

### 2.2 SleepStats methods

To quantify sleep-wake transitions using sleep-wake decision statistic data, the following steps are performed using the SleepStats Data Explorer Version 4 software:

1. File Import and Data Visualization
  • Load the raw data file containing feature vector feedback (featvecfb) into SleepStats by navigating to *File > Open* and selecting the appropriate featvecfb file. Once loaded, the software automatically plots the data for visual inspection and analysis.
2. Selection of Sleep-Wake Decision Statistic and Histogram
  • Using the panel at the top of the SleepStats program, select the *Sleep-Wake Decision Statistic* tab, providing a graphical time-series representation of the sleep-wake data for each animal. This visualization allows for temporal analysis of decision patterns.
  • Next, adjust the top panel to display the Sleep-Wake Decision Histogram, which provides a graphical representation of the frequency and distribution of sleep and wake decisions. This facilitates assessment of how well the decision threshold separated sleep and wake clusters.
3. Channel Selection for Distribution Validation
  • To ensure an appropriate distribution of sleep-wake data across all experimental channels, cages 1 through *x*, where *x* is the total number of channels analyzed, can be selected under the *Select Channel* tab for both the *Sleep-Wake Decision Statistic* and the *Sleep-Wake Decision Histogram.* This selection process ensures comprehensive data collection and validation across all animals in the study.
4. Exporting Sleep-Wake Decision Statistics
  • Under the *Export CSV* menu, select the option *Sleep-Wake Decision Statistic.* Specify the desired time interval for the analysis and use the *Export* function to generate a CSV file containing all relevant sleep-wake decision statistics.
  • The exported file, containing sleep-wake decision thresholds and decision statistics for each animal, can then be opened in Excel for further analysis.

### 2.3 Data processing: sleep-wake transitions

To systematically quantify sleep-wake transitions from the Sleep-Wake Decision Statistic datasets exported from the SleepStats software, we developed a custom Microsoft Excel™ VBA macro (CalculateSleepWakeTransitions). This macro automates the processing of high-resolution sleep-wake classification data, providing an efficient and reproducible method for quantifying transitions during fixed intervals across multiple animals (e.g., hours or days). The implementation optimizes data handling in large time-series datasets, ensuring structured, high-throughput analysis of sleep-wake fragmentation in rodents.

Each column in Sleep-Wake Decision Statistic datasets corresponds to a single rodent sleep-wake decision statistic, recorded every 2 s. The macro efficiently scans through the dataset, identifying transitions where a change in state occurs between consecutive time points. By iterating over the dataset, the macro calculates transition counts for each animal in predefined segments, producing structured outputs for downstream statistical analyses. Unlike standard Excel™ formulas, which become computationally expensive with large datasets, our VBA implementation minimizes execution time and memory load by leveraging vectorized processing and structured loops to optimize efficiency.

### 2.4 Macro functionality and execution

The Excel™ VBA macro initializes key parameters to ensure efficient processing of large data, including:

• Start Row (startRow): Specifies the first row containing numerical sleep-wake data
• Threshold Row (thresholdRow): Predefined threshold values
• Segment Size (SegmentSize): Defines the number of rows representing a segment of time (*i.e.,* 1 h)
• Column Range (cageStartCol, cageEndCol): Defines the start and end columns, corresponding to the individual animals

For each segment, the macro loops through the rows of data for each animal, comparing the current value to the previous row. If the threshold is crossed, the transition counter for that animal is incremented. Thresholds are dynamically assigned based on the values in the predefined row, allowing for customized analysis per animal. After all rows within a segment have been analyzed, the macro creates a new results worksheet, “TransitionsResults,” where the transition counts are stored. Each row in the TransitionsResults worksheet represents a specific segment of transitions, and each column corresponds to a separate animal. The process repeats for all segments in the dataset, ensuring systematic and organized transition recording.

### 2.5 Access and implementation

The macro code is open-source and available on GitHub at https://github.com/SIN-LAB-CU/SleepWakeTransitions. Users can copy the raw code and implement it in Excel™ by following these steps:

1. Open Excel™ and press *Alt + F11* to open the Visual Basic for Applications (VBA) editor.
2. Navigate to Insert > Module to create a new module.
3. Paste the macro code into the module, press save, and close the editor.
4. Return to Excel, press *Alt + F8*, select *CalculateSleepWakeTransitions* from the list, and click *Run.*

## 3 Example application

### 3.1 Animals and rigor

Animal studies were approved by the Institutional Animal Care and Use Committee at the University of Colorado Boulder (protocol 2819) and conducted in accordance with the National Institutes of Health guidelines for the care and use of laboratory animals. To evaluate sleep–wake transitions under normal physiological conditions, we analyzed transition counts in wild-type (C57BL/6J) male (n = 48) and female (n = 48) mice from six independent experimental groups (cohorts) recorded using two separate noninvasive piezoelectric cage systems. All mice were bred in-house from breeder pairs obtained from The Jackson Laboratory (Bar Harbor, ME). Mice were singly housed and maintained on a 12-h light:dark cycle (200 lux, cool white, fluorescent light) at an ambient temperature of 24 °C ± 2 °C. All mice were acclimated to sleep cages for a minimum of 5 days prior to initiation of data collection. Mice were fed a normal diet of standard rodent chow, and food and water were available *ad libitum*. Transitions were quantified on an hourly basis (*i.e.,* hour segments) to assess sex differences in sleep continuity over a 24-h period.

### 3.2 Statistical analysis

Using the glmmTMB package in the R statistical computing environment (Brooks et al., 2017; Team, 2025), we fit hierarchical generalized linear mixed models to the hourly number of transitions and the total number of transitions datasets (Stroup, 2012; Faraway, 2016). For both outcomes, we specified negative-binomial response distributions because the data exhibited overdispersion (Hilbe, 2014).

In the model for hourly transitions, we included a two-way fixed effects interaction between sex and a nonlinear Zeitgeber (ZT) time effect; nonlinearity in ZT was modeled with a basis spline with three degrees of freedom (Mannino et al., 2024; Perperoglou et al., 2019). In the model for total transitions, we specified a two-way fixed-effects interaction between sex and period (light vs. dark). Both models included random intercepts for individual animals nested within their respective cohort, thereby directly accounting for the repeated measures within animals and clustering of animals recorded during different experimental periods (Mannino et al., 2024, 2025).

Inferences were based on model coefficient estimates (β), estimated conditional means with 95% confidence intervals, and corresponding p-values (Mannino et al., 2024, 2025). To evaluate consistency of the approach, we obtained the model-estimated conditional variance among cohorts and cohort-specific random intercepts. Hypnograms for representative animals were generated using Python (Matplotlib), with sleep and wake states displayed as binary time series (sleep = 1, wake = 0).

### 3.3 Results

#### 3.3.1 Sleep-wake transitions differ by sex across the light-dark cycle

**Fig. 3:**
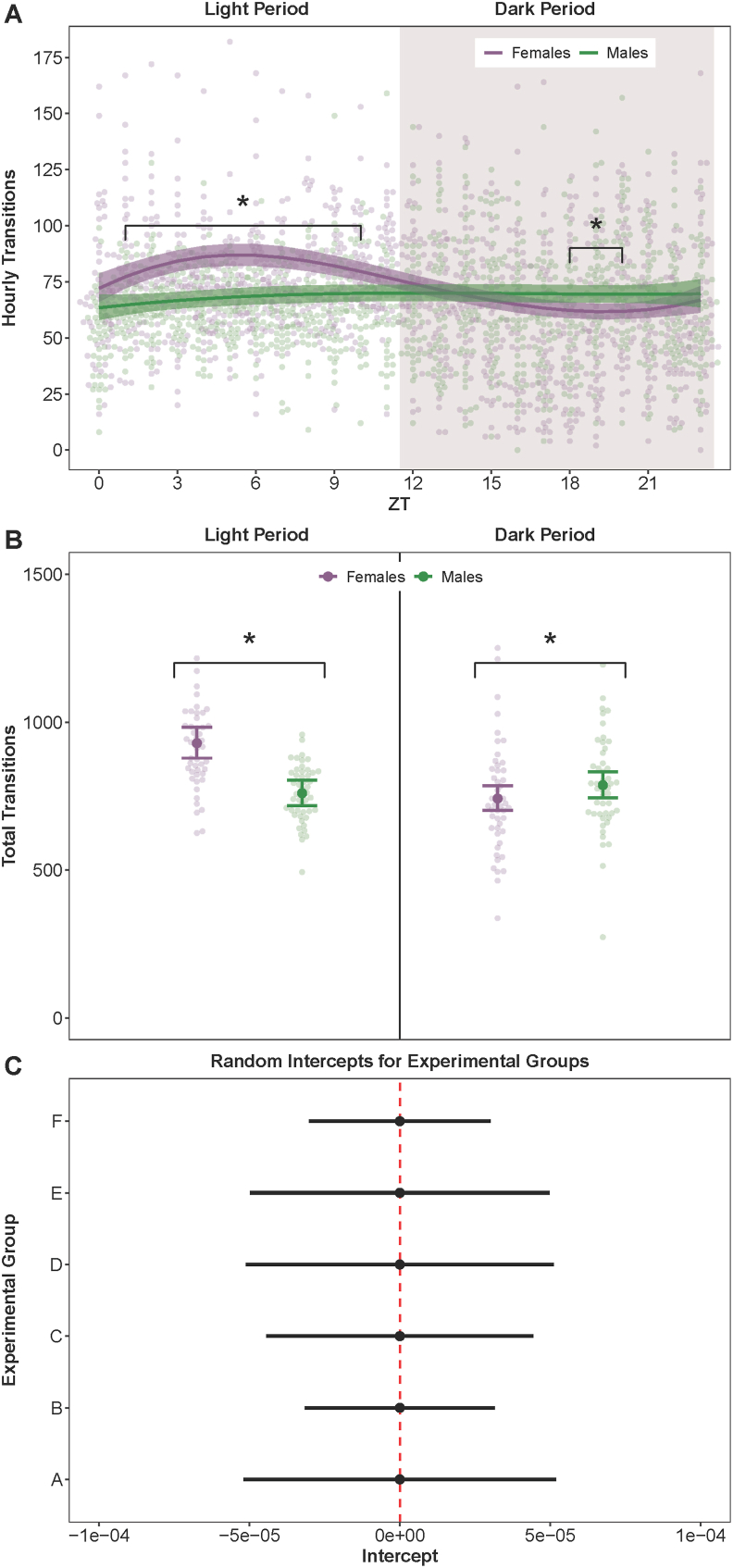
**Sleep-Wake Transitions in Wild-Type Male and Female Mice Over a 24-Hour Period.** The number of sleep-wake transitions per hour was assessed in wild-type male and female mice using a noninvasive piezoelectric cage system. Hourly transitions (**A**) and total transitions (**B**) were analyzed during the light and dark periods using generalized linear mixed effects models with negative binomial response distributions. Female mice exhibited significantly more transitions per hour than males during the light period. Negligible variation in the transitions data (**C**) was attributed to animals belonging to six independent experimental groups (cohorts). Results are presented as model-estimated conditional mean point estimates (solid lines in **A**, large dots in **B** and **C**) and 95% confidence intervals (shaded bands in **A**, error bars in **B**, horizontal black lines in **C**); the small color-coded background dots in **A** and **B** denote raw data observations. ∗ Indicates *p* < 0.05.

Significantly different number of transitions per hour existed between sexes across multiple time windows, suggesting dissimilar sleep fragmentation under baseline conditions (Fig. 3A). During the light period, female mice exhibited significantly more hourly transitions than males between 1 and 10 ZT (β = 0.52, 95% CI = 0.29–0.75, *p* = 0.000008). Transitions per hour were also greater for females than males during the first (ZT = 0) and last (ZT = 11) hours of the light period, but the differences were not statistically significant (β = 0.17, 95% CI = −0.005–0.34, *p* = 0.06). When considering total transitions across the entire light period (Fig. 3B), females exhibited substantially more transitions than males (β = 0.20, 95% CI = 0.12–0.28, *p* = 0.0000006).

During the dark period, females had significantly fewer hourly transitions than males from 18 to 20 ZT (β = −0.44, 95% CI = −0.77–0.10, *p* = 0.0101; Fig. 3A). However, no significant differences between sexes were observed at other ZT during the dark period (*p* = 0.2228). The total number of transitions during the dark period significantly differed between sexes, with males having a greater number of transitions (β = 0.26, 95% CI = 0.15–0.37, *p* = 0.000005; Fig. 3B). The model-estimated conditional variance among cohorts was negligible (σ^2^ = 2.62 × 10^−10^) and the point estimates of random intercepts were functionally identical among all cohorts, with substantially overlapping 95% CIs (Fig. 3C).

#### 3.3.2 Representative hypnograms support sex differences in sleep fragmentation

**Fig. 4:**
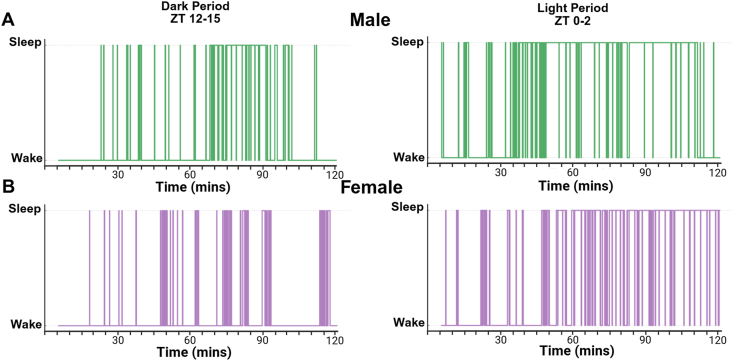
**Hypnogram showing sleep-wake transitions from a male and female mouse.** A hypnogram was generated from a representative male and female mouse where each hypnogram represents 2 h of sleep-wake data. Data are shown for **(A)** male and **(B)** female mice during the dark period (ZT 12–15) and light period (ZT 0–2). ZT = Zeitgeber Time.

Hypnograms indicate that sex differences in sleep-wake transitions are most pronounced during the light period, when sleep is typically more consolidated in mice (Fig. 4). In contrast, during the dark period, males and females exhibit more similar transition patterns, except for differences in specific time windows. These plots illustrate the utility of transition-based metrics for detecting temporal patterns in sleep fragmentation.

## 4 Discussion

We present a novel tool and associated analytical approach for quantifying sleep-wake transitions in laboratory rodents from activity data recorded noninvasively using piezoelectric cage systems. This method provides an objective measure of sleep continuity and fragmentation from movement and respiration data collected by piezoelectric sensors, and our analysis demonstrated the approach has exceptionally high consistency among experimental groups and cage systems. Thus, our method represents a reliable, scalable, and reproducible noninvasive alternative to invasive EEG/EMG-based assessments, enabling detailed characterization of sleep architecture without the need for surgical implantation or manual scoring.

Compared to conventional metrics obtained from noninvasive methods, which rely on total sleep duration or mean bout length, our method extracts transitions as discrete, biologically meaningful events. The transition-based metric complements existing sleep analysis tools by directly capturing sleep instability and fragmentation, which are features increasingly recognized as important in models of neurodegeneration, injury, and circadian disruption. Our example application demonstrates that this approach accurately replicated known sex differences in sleep continuity, supporting its utility in detecting biologically relevant variation in preclinical neuroscience studies (Mannino et al., 2024).

The piezoelectric system we used has been validated to achieve >90% concordance with EEG-based classification (Mang et al., 2014), and its 2-s resolution allows for granular analysis without requiring subjective definitions of bout length or state transitions. Although EEG-based scoring remains the de facto ‘gold standard,’ the piezoelectric system offers an efficient, high-throughput, noninvasive alternative that is well-suited for large-scale or longitudinal studies. While validated against EEG-based scoring, additional validation may further refine classification thresholds and improve accuracy. We note that, although our study focuses on the piezoelectric platform, the transition-analysis framework we developed could be adapted for other noninvasive systems (e.g., video) and species where immobility-based sleep scoring is used. Validation across modalities is an important future direction.

In addition to its utility in baseline conditions, this method is readily adaptable to studies examining circadian phase shifts, pharmacological modulation, aging, or disease-related changes in sleep architecture. Future enhancements could involve machine learning integration to improve transition detection or the inclusion of additional physiological signals, such as body temperature or activity rhythm data, to enrich the analysis. Overall, the combination of the VBA macro and analysis pipeline provides a robust and accessible framework for evaluating sleep continuity in rodent models using piezoelectric cage systems, thereby addressing a growing need for standardized, noninvasive tools in neuroscience research.

## 5 Conclusion

Sleep-wake transitions offer a powerful yet underutilized lens for assessing sleep continuity and fragmentation, which are key dimensions of sleep architecture with direct relevance to neuroscience. By developing a standardized and reliable transition-based analysis pipeline for use with noninvasive piezoelectric cage systems, we provide a practical and scalable method for quantifying sleep quality in rodent models. The approach is efficient, reproducible, and sensitive to biologically meaningful differences, such as sex-specific variation in fragmentation patterns. Its applicability across experimental paradigms, including those involving injury, neurodegeneration, circadian disruption, and pharmacological interventions, makes it a valuable addition to the methodological toolkit for sleep and systems neuroscience. As the field continues to embrace high-throughput, translationally relevant tools, this method fills a critical gap between duration metrics and invasive EEG-based assessments, enabling rigorous, noninvasive evaluation of sleep-wake dynamics in a wide range of preclinical studies.

## Author contributions

RR, GM, and MO conceptualized the study. RR and GM executed the mouse studies. GM and AL developed the method for transition analysis. SMM analyzed all data and completed statistical analyses and visualization of the data. MO helped with interpretation of results. All authors contributed to the writing and editing of this manuscript. RR and MO obtained funding for this work. RR and MO supervised all experiments.

## Data availability statement

The macro code is open-source and available on GitHub at https://github.com/SIN-LAB-CU/SleepWakeTransitions. Users can copy the raw code and implement it in Excel.

## Author contributions (CRediT)

Conceptualization: R.K. Rowe, G.S. Mannino, M.R. Opp.

Methodology: G.S. Mannino, A. Lugo, R.K. Rowe.

Investigation: G.S. Mannino, R.K. Rowe.

Software: G.S. Mannino, A. Lugo.

Formal analysis: S.M. Murphy.

Validation: S.M. Murphy, R.K. Rowe, Visualization: S.M. Murphy, Resources: R.K. Rowe, M.R. Opp.

Supervision: R.K. Rowe, M.R. Opp.

Funding acquisition: R.K. Rowe, M.R. Opp.

Writing – original draft: R.K. Rowe, G.S. Mannino, S.M. Murphy.

Writing – review & editing: All authors.

## Ethics approval statement

All animal procedures were conducted in accordance with the guidelines of the University of Colorado Boulder Institutional Animal Care and Use Committee (IACUC) and were approved under protocol #2819. Experimental data are reported in compliance with the ARRIVE guidelines to ensure rigorous and transparent reporting of animal research.

## Funding sources

This work was supported, in part, by grants from the Department of Defense awards W81XWH-22-1-083 (RR) and W81XWH-22-1-0384 (MO). RR and GM were supported, in part, by funds from the Department of Integrative Physiology at the University of Colorado Boulder, and the National Institutes of Health (NIH)-NS132672. AL was supported by the National Institutes of Health (NIH)-T32-HL149646.

## Declaration of competing interest

The authors have no disclosures.
