# Supplementary material for: A tool for high-throughput quantification of sleep-wake transitions in data from noninvasive piezoelectric cage systems
Source: Curr Res Neurobiol. 2026 Jan 30;10:100156. doi: 10.1016/j.crneur.2026.100156 (PMC12907240; doi:10.1016/j.crneur.2026.100156)

**SleepStats Methods**

1. File Import and Data Visualization


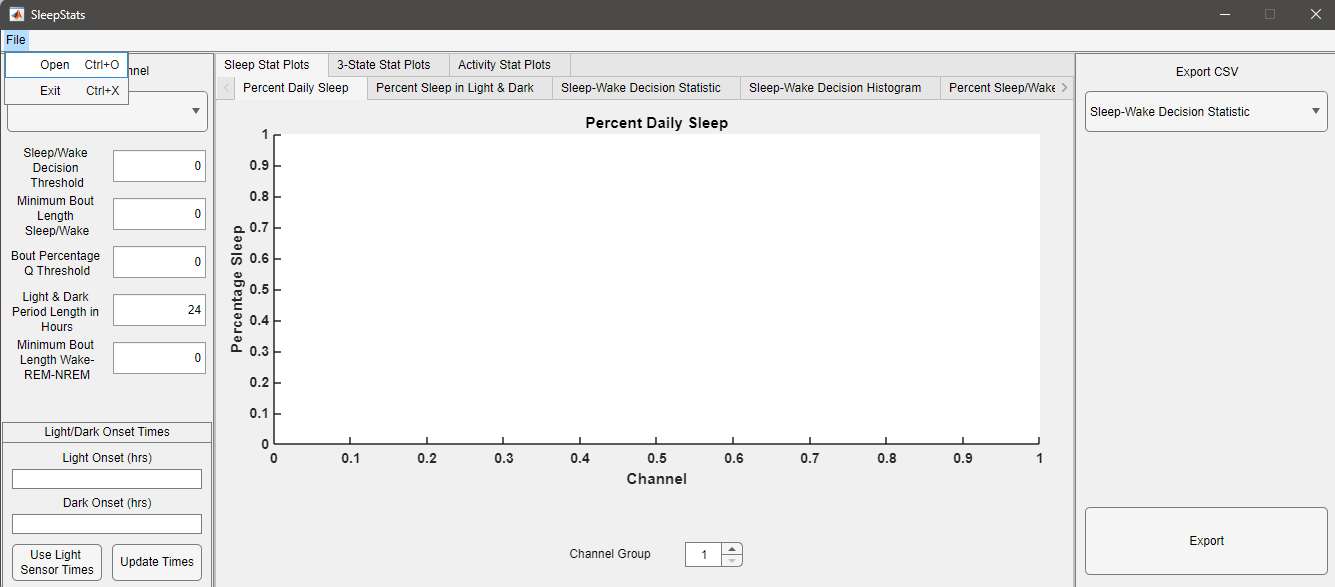


- Load the raw data file containing feature vector feedback (featvecfb) into SleepStats by navigating to *File > Open* and selecting the appropriate featvecfb file. Once loaded, the software automatically plots the data for visual inspection and analysis.

1. Selection of Sleep-Wake Decision Statistic and Histogram


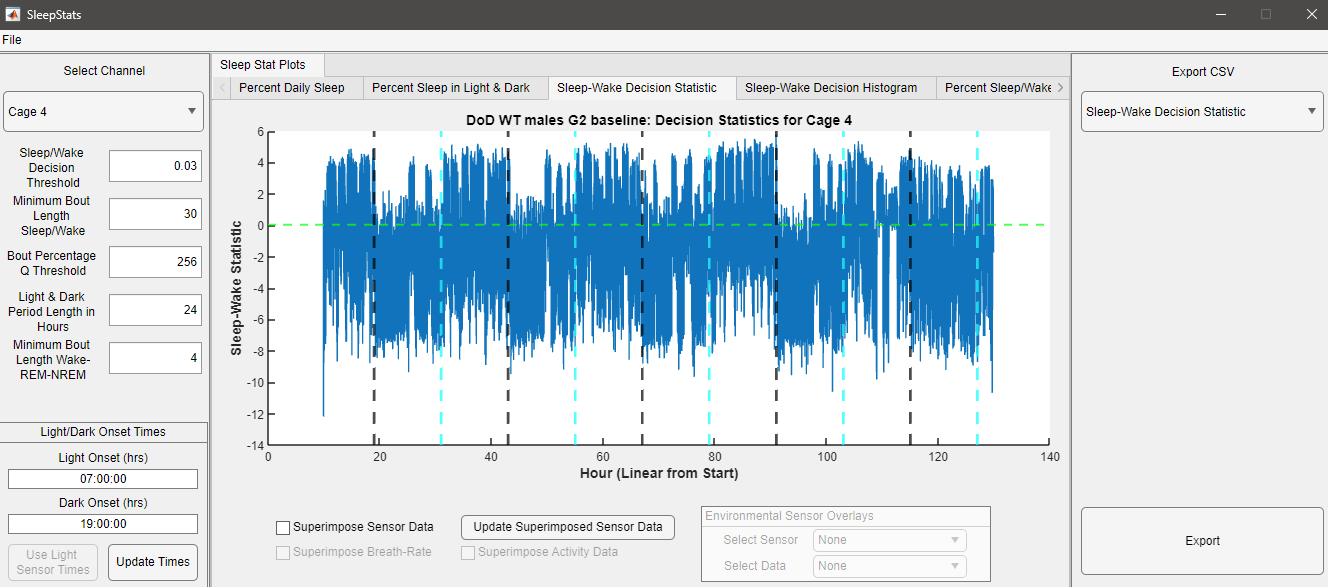


- Using the panel at the top of the SleepStats program, select the *Sleep-Wake Decision Statistic* tab, providing a graphical time-series representation of the sleep-wake data for each animal. This visualization allows for temporal analysis of decision patterns.


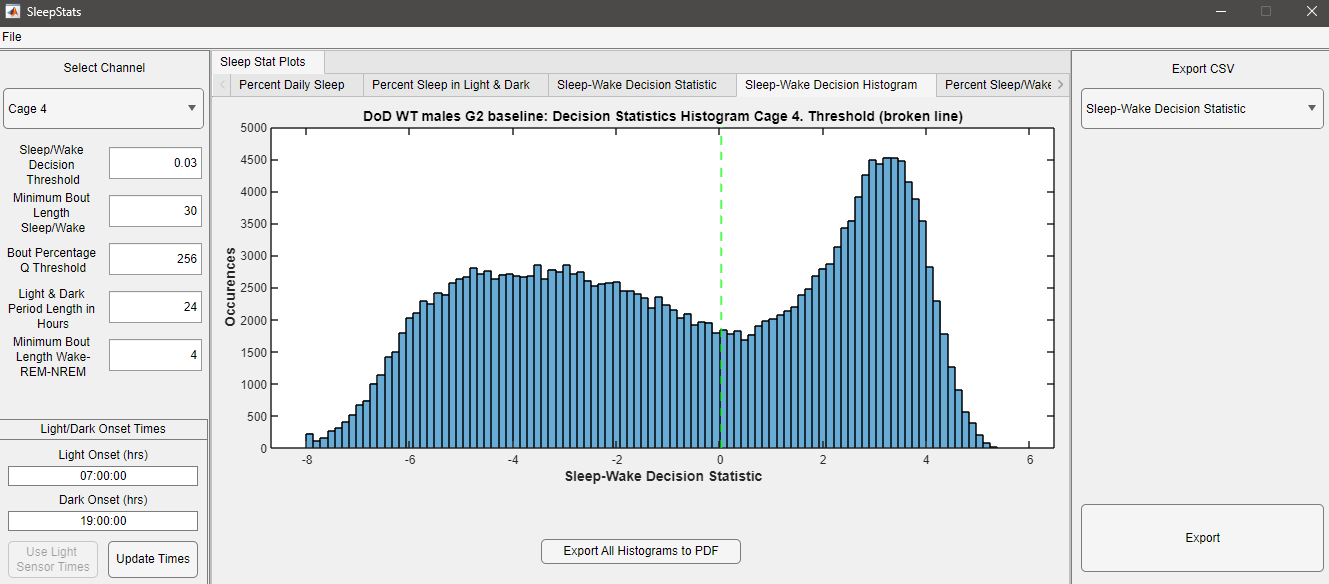


- Next, adjust the top panel to display the Sleep-Wake Decision Histogram, which provides a graphical representation of the frequency and distribution of sleep and wake decisions. This facilitates assessment of how well the decision threshold separated sleep and wake clusters.

1. Channel Selection for Distribution Validation


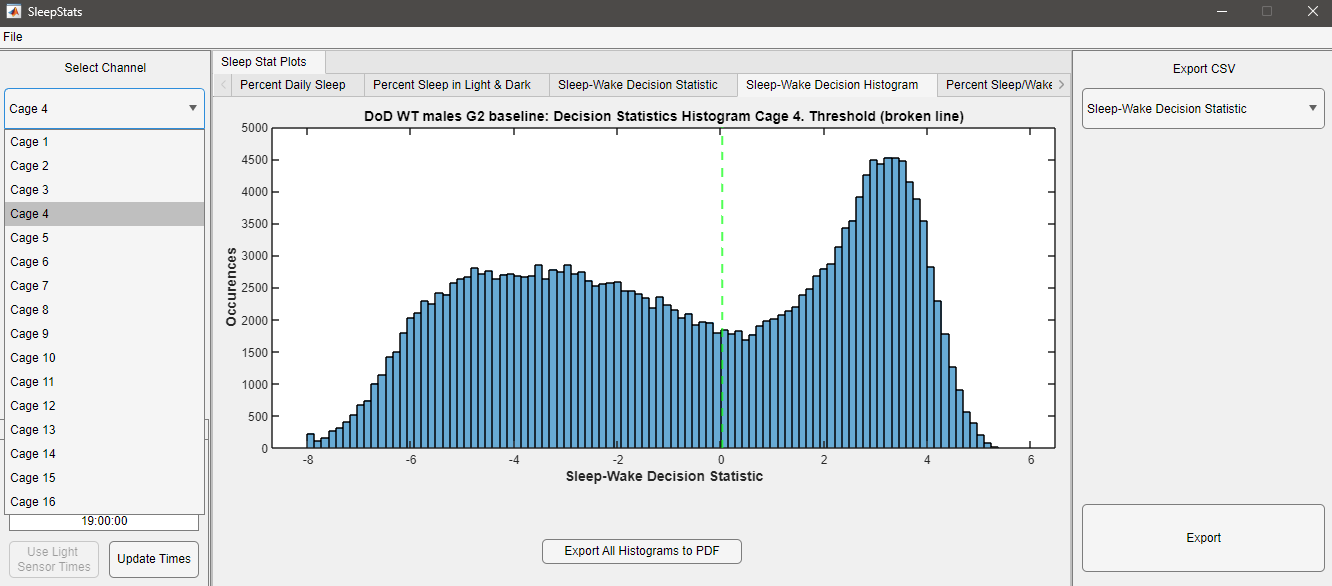


- To ensure an appropriate distribution of sleep-wake data across all experimental channels, cages 1 through (*x*), where ‘*x*’ is the total number of channels analyzed, can be selected under the *Select Channel* tab for both the *Sleep-Wake Decision Statistic* and the *Sleep-Wake Decision Histogram.* This selection process ensures comprehensive data collection and validation across all animals in the study.

1. Exporting Sleep-Wake Decision Statistics


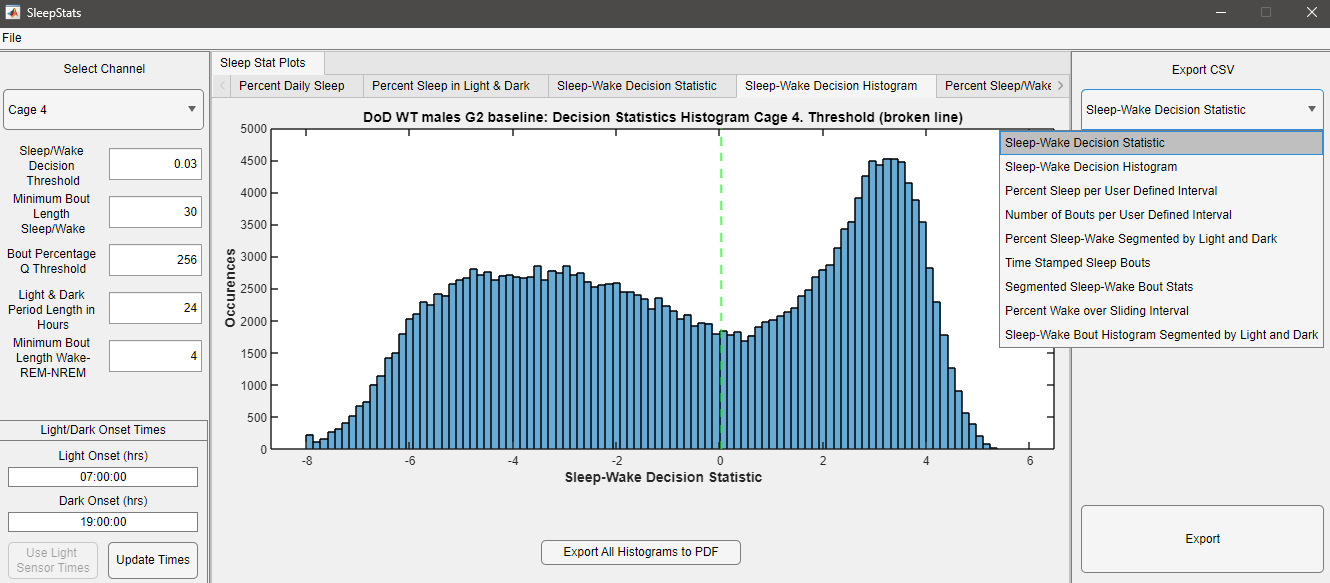


- Under the *Export CSV* menu, select the option *Sleep-Wake Decision Statistic*. Specify the desired time interval for the analysis and use the *Export* function to generate a CSV file containing all relevant sleep-wake decision statistics.
- The exported file, containing sleep-wake decision thresholds and decision statistics for each animal can then be opened in Excel for further analysis.

**Data Processing: Sleep-Wake Transitions**

1. Open Excel and press *Alt + F11* to open the Visual Basic for Applications (VBA) editor


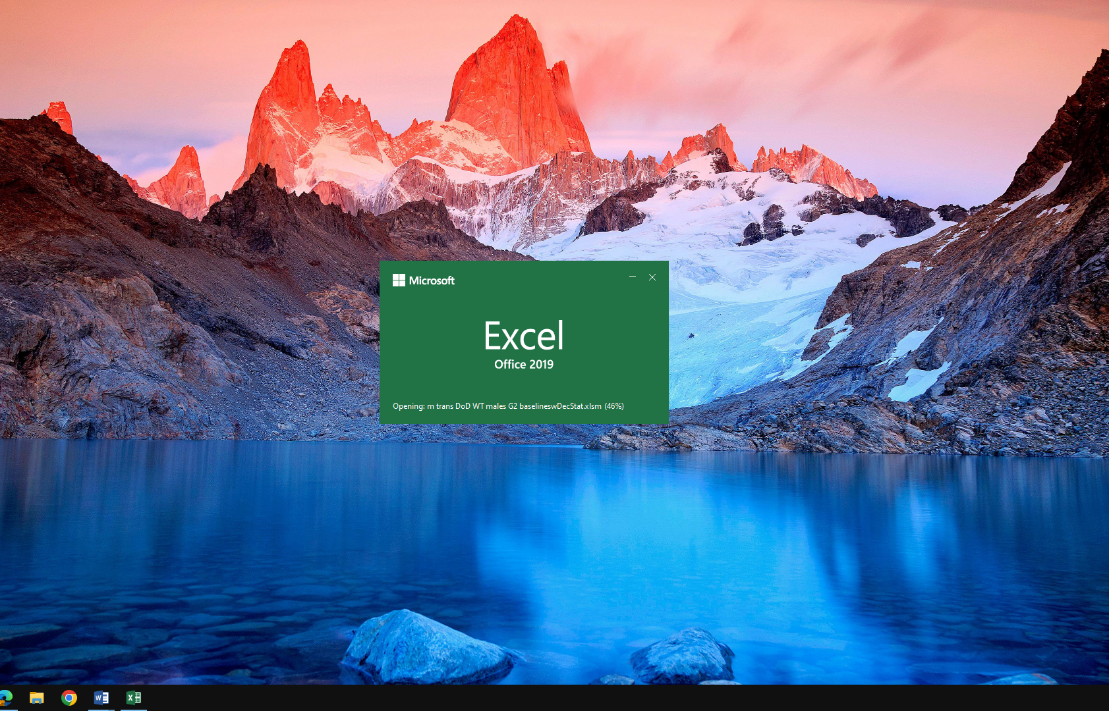


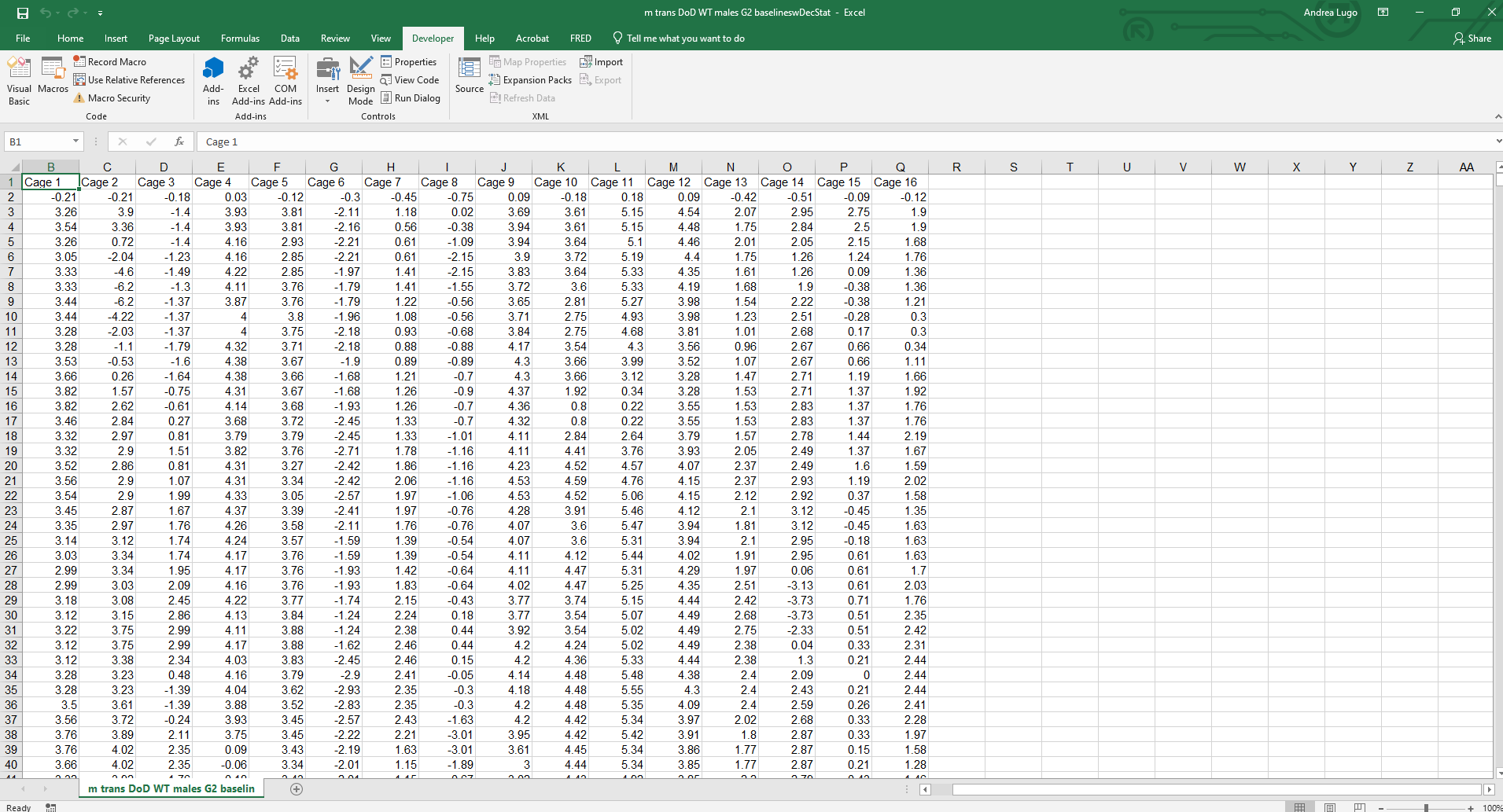


1. Navigate to Insert > Module to create a new module


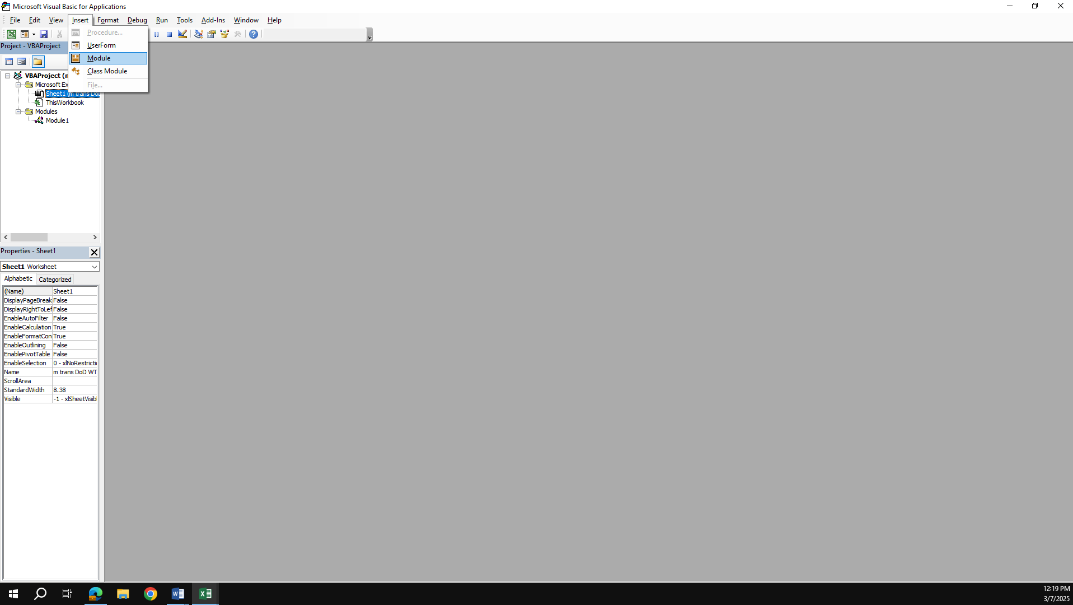


The macro code is open-source and available on GitHub at <https://github.com/SIN-LAB-CU/SleepWakeTransitions>. Users can copy the raw code and implement it in Excel™ by following these steps:

1. Open Excel™ and press *Alt + F11* to open the Visual Basic for Applications (VBA) editor.
2. Navigate to Insert > Module to create a new module.
3. Paste the macro code into the module, press save and close the editor.
4. Return to Excel, press *Alt + F8*, select *CalculateSleepWakeTransitions* from the list, and click *Run*.

Paste the macro code into the module save it and close the editor.


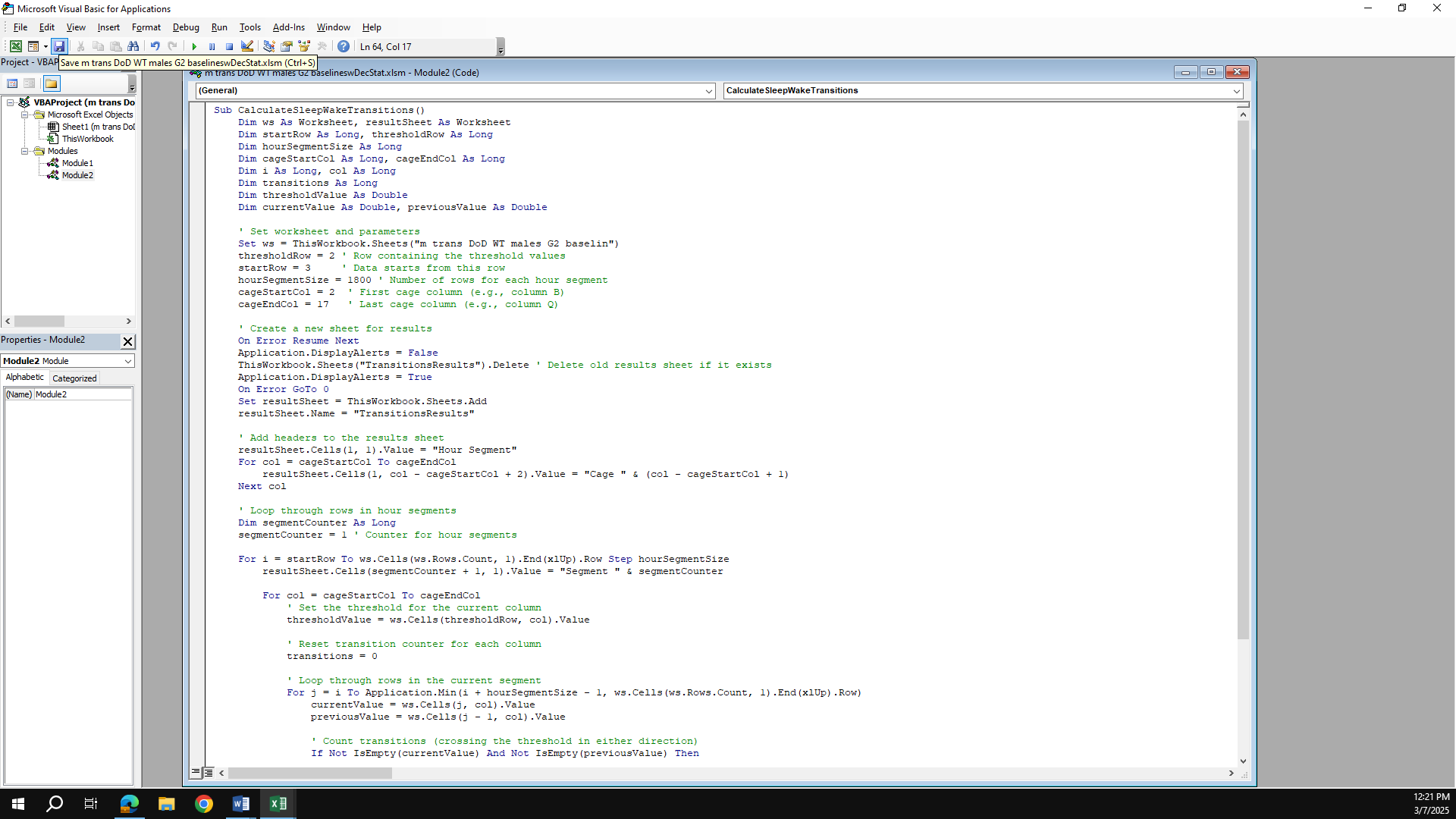


1.
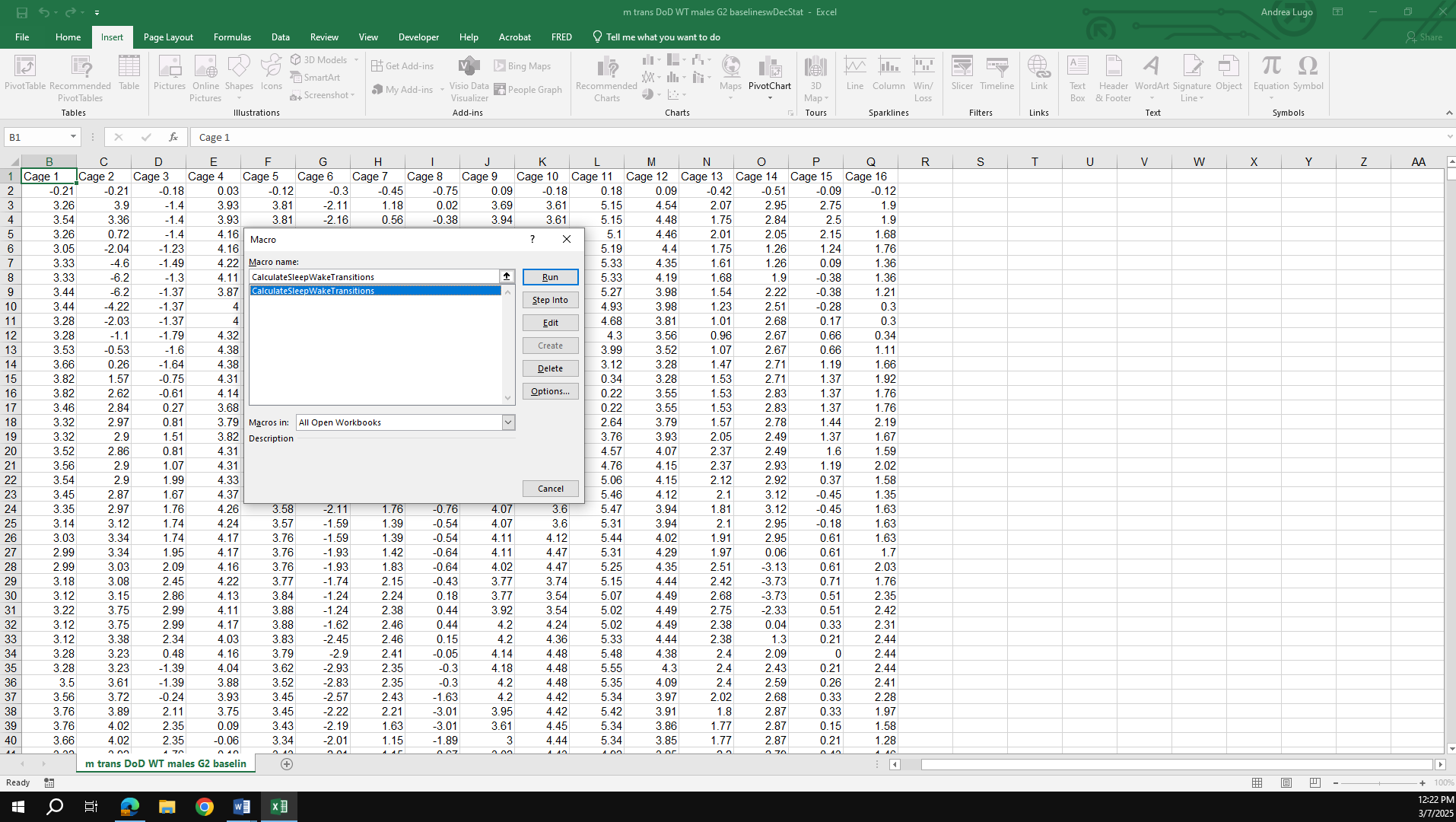
Return to Excel, press *Alt + F8*, select *CalculateSleepWakeTransitions* from the list, and click *Run*.
2. Your transitions results are now ready.


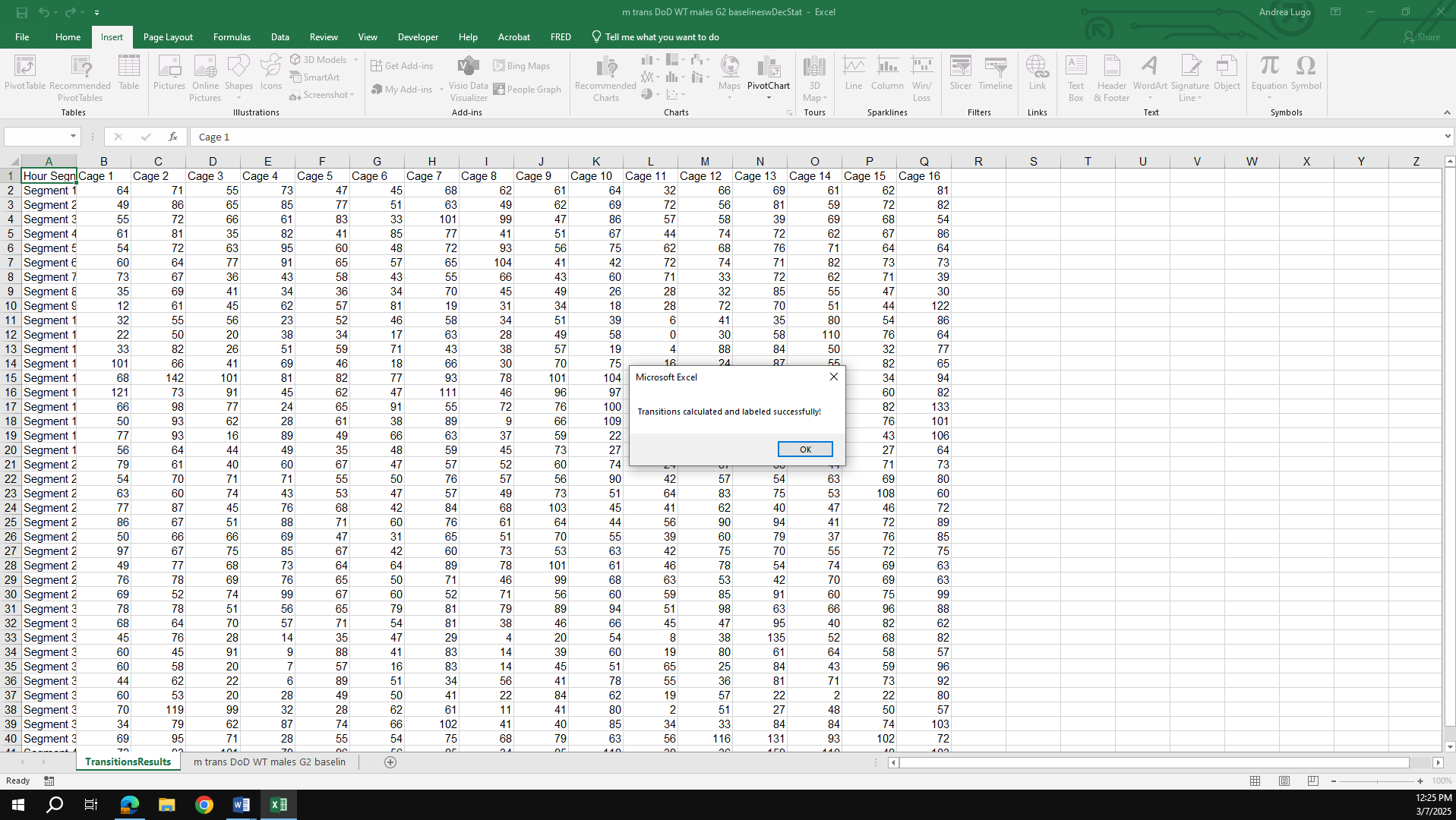

Supplement: Multimedia component 1 [file mmc1.docx]
